# Prehospital Trauma Scoring Systems for Evaluation of Trauma Severity and Prediction of Outcomes

**DOI:** 10.3390/medicina59050952

**Published:** 2023-05-15

**Authors:** Radojka Jokšić-Mazinjanin, Nikolina Marić, Aleksandar Đuričin, Zoran Gojković, Velibor Vasović, Goran Rakić, Milena Jokšić-Zelić, Siniša Saravolac

**Affiliations:** 1 Department of Emergency Medicine, Medical Faculty, University of Novi Sad, 21000 Novi Sad, Serbia; aleksandar.djuricin@mf.uns.ac.rs (A.Đ.); goran.rakic@mf.uns.ac.rs (G.R.); 2 Institute for Emergency Medical Services Novi Sad, 21000 Novi Sad, Serbia; maric1992@gmail.com (N.M.); sinisa.saravolac@outlook.com (S.S.); 3 Department of Surgery, Medical Faculty, University of Novi Sad, 21000 Novi Sad, Serbia; zoran.gojkovic@mf.uns.ac.rs; 4 Clinic for Orthopedic Surgery and Traumatology, University Clinical Center of Vojvodina, 21137 Novi Sad, Serbia; 5 Department of Pharmacology, Toxicology and Clinical Pharmacology, Medical Faculty, University of Novi Sad, 21000 Novi Sad, Serbia; velibor.vasovic@mf.uns.ac.rs; 6 Department of Intensive Surgical Therapy, Institute for Child and Youth Health Care Vojvodina, Pediatric Surgery Clinic, 21000 Novi Sad, Serbia; 7 Emergency Medical Service, Health Centre Bečej, 21220 Bečej, Serbia; milenajoksiczelic@gmail.com

**Keywords:** injury, trauma, trauma scoring systems, indicators of severity of injury, triage, emergency medical services

## Abstract

Introduction: Trauma scoring systems in prehospital settings are supposed to ensure the most appropriate in-hospital treatment of the injured. Aim of the study: To determine the sensitivity and specificity of the CRAMS scale (circulation, respiration, abdomen, motor and speech), RTS score (revised trauma score), MGAP (mechanism, Glasgow Coma Scale, age, arterial pressure) and GAP (Glasgow Coma Scale, age, arterial pressure) scoring systems in prehospital settings in order to evaluate trauma severity and to predict the outcome. Materials and Methods: A prospective, observational study was conducted. For every trauma patient, a questionnaire was initially filled in by a prehospital doctor and these data were subsequently collected by the hospital. Results: The study included 307 trauma patients with an average age of 51.7 ± 20.9. Based on the ISS (injury severity score), severe trauma was diagnosed in 50 (16.3%) patients. MGAP had the best sensitivity/specificity ratio when the obtained values indicated severe trauma. The sensitivity and specificity were 93.4 and 62.0%, respectively, for an MGAP value of 22. MGAP and GAP were strongly correlated with each other and were statistically significant in predicting the outcome of treatment (OR 2.23; 95% Cl 1.06–4.70; *p* = 0.035). With a rise of one in the MGAP score value, the probability of survival increases 2.2 times. Conclusion: MGAP and GAP, in prehospital settings, had higher sensitivity and specificity when identifying patients with a severe trauma and predicting an unfavorable outcome than other scoring systems.

## 1. Introduction

Approximately 1.3 million people die each year as a result of road traffic crashes (73% of victims are male). About 93% of road traffic deaths occur in low- and middle-income countries. Road traffic injuries are the leading cause of death for children and young adults aged 5–29 years [1]. Taking into consideration other types of trauma resulting in fatalities, it can be concluded that trauma is the leading cause of death in people aged 5–24 years globally and of disability for people aged 5–44 years. [2]. Trauma is the fifth leading cause of death in the elderly population. Although injuries are usually less severe in older people, the risk of complications is increased and the percentage of fatal outcomes rises. For the same trauma score value, older people have a higher probability of death. Comorbidities, especially cardiovascular ones, are thought to be the main cause. Other important causes include the use of medicine, including oral anticoagulants, and the reduced functional reserves of the body of older people [3]. Trauma severity depends on the impact of the traumatic forces on the human body. Most trauma patients present with minor to moderate injuries. Only 5% of patients have critical injuries, while urgent surgical intervention is necessary in 10–15% patients [4,5].

Death caused by trauma has a trimodal distribution in undeveloped and developing countries, compared to developed countries, which have a bimodal distribution of trauma mortality. The first peak of mortality in all countries, especially in those that are undeveloped, is in the first hour after the trauma (immediate death). In undeveloped countries, the second peak of mortality occurs in the first four hours (early death). Developed countries, due to the improvements in the emergency medical services and collaboration between the prehospital and hospital levels of the health system, have managed to decrease the rates of immediate and early deaths, which has led to a decrease in total post-traumatic mortality. Late death occurs several days after trauma and occurs due to multiple organ dysfunction or sepsis [6,7].

The quality of prehospital trauma triage is dependent on the accuracy of and compliance with the trauma score. Trauma scoring systems play an important role in the improved management of trauma patients and can decrease mortality and disability rates. By using trauma scoring systems, it is possible to identify patients with critical trauma severity and alert all parts of the health system. The ultimate goal should be to improve trauma management and increase survival rates in patients with critical trauma [8,9].

Different scoring systems (anatomical, physiological, and combined) are used to evaluate the severity of the trauma and to predict the outcome of treatment for the injured patient. The most accurate predictor of trauma mortality is the anatomical ISS score (injury severity score). Its disadvantage is that it assesses trauma severity based on the anatomical criteria of injury severity, which can only be obtained after a completed diagnosis or autopsy. Therefore, it is not suitable for the prehospital and initial hospital levels. The most frequently used trauma scoring systems at the prehospital level are physiological, of which RTS is the most common. The revised trauma score (RTS), unlike the previously mentioned anatomical scores, is easily measured using the GCS (Glasgow Coma Scale), systolic blood pressure (SBP), and respiratory rate (RR), but its reliability is questionable. It is most important to develop a scoring system for use during the “golden hour” that will most accurately predict the outcome for the trauma patient in order to take the best treatment measures. Therefore, there is a need to design new trauma scoring systems and test their reliability [10,11].

The goal of this scientific research was to determine the sensitivity and specificity of the CRAMS scale (circulation, respiration, abdomen, motor and speech), the RTS score, and the MGAP (mechanism, GCS, age, SBP), and GAP (GCS, age, SBP) scores utilized during the prehospital stage in order to assess the severity of trauma and their possible application in the prediction of survival in trauma patients. Additionally, we aimed to determine which of the scores is an independent predictor when assessing the severity of trauma and predicting the survival rate of injured patients. 

## 2. Materials and Methods

### 2.1. Territory Covering the Study and Management of the Emergency Department

The Emergency Medical Services (EMS) of the city of Novi Sad cover the municipalities of Novi Sad and Sremski Karlovci, with a total population of 383,997 inhabitants according to the 2022 census. There are eight EMS teams, each consisting of a medical doctor, medical technician, and a driver. Calls for intervention are distributed through the EMS Dispatching Centre.

The EMS teams of the Health Centre in Bečej cover the municipality of Bečej, with a population of 31,082 inhabitants according to the 2022 census. There is only one EMS team on duty, consisting of a medical doctor and medical technician, while a driver from the Health Centre covers emergency call-outs. There is no centralized Dispatching Centre and the calls are answered by a doctor on duty.

All trauma patients from both territories were transported to the Emergency Centre in Novi Sad, which is a part of the University Clinical Centre of Vojvodina. 

### 2.2. Study Design and Data Collection Procedure

A prospective observational study was conducted. The study group consisted of trauma patients older than 18 years and prehospitally treated by the EMS of the city of Novi Sad and municipality of Bečej, who were transferred to the Emergency Centre in Novi Sad. Patients with minor injuries and who were not transferred to the Emergency Centre by EMS teams were excluded from this study. In addition to this, the study excluded all those patients who decided to transport themselves to the Emergency Centre after initial treatment by the EMS teams, those patients who were declared dead at the road accident site, and hospitalized patients who were transferred to any other medical institution. The consent of the Ethics Committee of EMS Novi Sad (protocol code No1243, 17 September 2018), the Ethics Committee of Becej Health Center (protocol code No16027, 14 October 2018) and the Ethics Committee of the University Clinical Center of Vojvodina (protocol code No 894/2018, 24 December 2018.) was obtained to conduct this study. Every patient who participated in this study signed an informed consent form. For patients who were unable to understand and sign the consent form, consent was given by relatives. Data were collected prospectively.

For every patient, after the initial trauma management, a questionnaire was filled in by a prehospital doctor, including personal data, vital parameters, GCS, oxygen saturation in peripheral blood measured by pulse oximeter (SaO2, heart rate (HR), RR, SBP), treatment times, mechanism of injury, and assessment of trauma severity without the use of trauma scores. The doctors involved in the prehospitalization management of injured patients filled out paper questionnaires, which they then handed over to the researchers. Based on the vital parameters obtained prehospitalization, the results of the trauma scoring systems were calculated by the researchers, including the RTS score for triage (tRTS) to evaluate the trauma severity and the prediction RTS to predict the outcome, the CRAMS scale, and the MGAP and GAP scores. The ISS was used to objectively assess the trauma severity and was calculated after discharge from the University Clinical Centre of Vojvodina and Institute for Pulmonary Diseases of Vojvodina or after an autopsy at the Institute for Forensic Medicine of the University Clinical Centre of Vojvodina. A threshold ISS ≥ 15 was used to classify major trauma, and an ISS ≤ 15 was used to classify minor injury [12]. In addition to the severity of the injury markers, the outcome of the treatment, from survival to hospital discharge, was also observed. 

### 2.3. Statistical Analysis

The results were processed using the Statistical Package for Social Sciences–SPSS 21.

Numerical variables were presented as the mean values, value ranges, and standard deviation, and categorical variables were presented as frequencies and percentages. Numerical attributes were compared by using Student’s *t*-test and the nonparametric Mann–Whitney U test, depending on the data distribution. The χ2 test and Fisher’s exact test were used to determine the frequency differences of categorical variables. The ROC (receiver operating characteristic) curve was used to test the sensitivity and specificity of the trauma scores. The ROC curve is a graphic representation of the sensitivity and specificity of trauma scores at limit values. Results were represented graphically using the coordinate system (sensitivity values on the ordinate axis and specificity values on the abscissa). Minor trauma was taken as a reference value. 

Univariate regression analysis was used to determine the impact of trauma scores on minor injuries and survival rates during the period of hospitalization. Statistically significant trauma scores were included in a multivariate regression analysis with the aim to determine the correlation between trauma severity and outcome. Variables with associated *p* values < 0.05 were considered statistically significant.

## 3. Results

A total of 307 trauma patients were enrolled in the study. According to the ISS, 50 patients had major trauma and 257 patients had minor trauma. The average ISS of the patients with major and minor trauma was 25.8 ± 9.0 and 5.6 ± 3.3, respectively (Mann–Whitney test: U = 0.00; *p* = 0.000). ISS was considerably lower in major trauma patients who died during hospitalization: 40.7 ± 6.6 (Mann–Whitney test: U = 12.00; *p* = 0.000). 

Values of GKS were statistically lower in the group of patients with severe trauma (12.0 ± 3.6), especially for patients that did not survive until hospital discharge (6.6 ± 3.4). Similar results were obtained during prehospital measurement of SaO2. For the patients that did not survive until hospital discharge, the average arterial oxygen saturation was 75.0 ± 20.3%. (Table 1). SBP 100–120mm Hg was measured in 52.0% of severe trauma patients, and 62.5% of those who did not survive until hospital discharge had SBP values measuring under 100 mmHg. The most common mechanism of trauma identified in all patients was blunt impact injury, except for the patients that did not survive until hospital discharge (62.5%).

EMS doctors treating the patients during field response were asked to determine the severity of trauma without using trauma scores. They misdiagnosed minor trauma in 42% of patients with major trauma and 12.5% of patients that did not survive until hospital discharge.

Based on the prehospital vital parameters, four types of trauma score were calculated: tRTS and prediction RTS, CRAMS scale, MGAP, and GAP. All calculated trauma scores were significantly lower in patients with major trauma, especially in the group of hospitalized patients with a fatal outcome (Table 2). 

**Figure 1:**
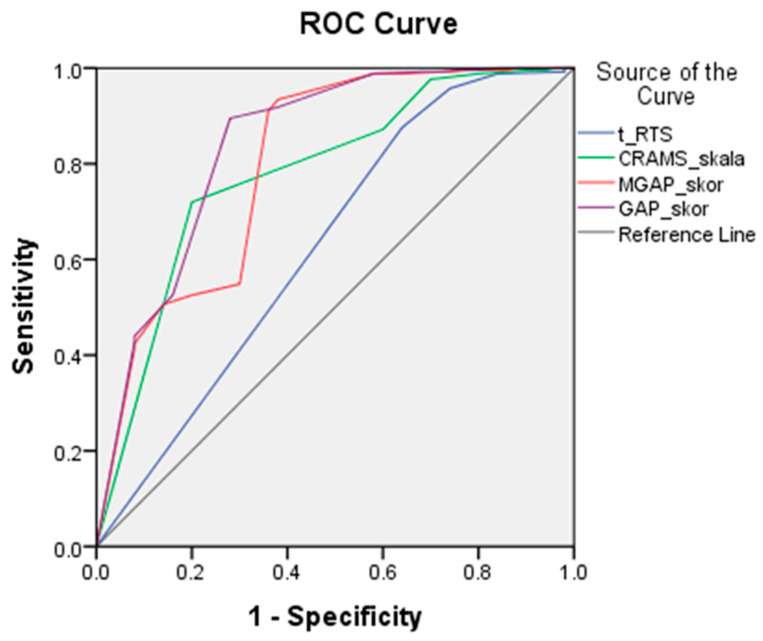
ROC (receiver operating characteristic) curve of trauma scores measured prehospitalization in relation to trauma severity. T-RTS score—revised trauma score for triage; CRAMS scale—circulation, respiration, abdomen, motor, and speech; MGAP—mechanism, Glasgow Coma Scale, age, systolic blood pressure; GAP—Glasgow Coma Scale, age, systolic blood pressure.

From Table 3 and Figure 1, we can see that the ability of the MGAP and GAP scores to distinguish light from severe injury was very good and that GAP (AUC = 0.842) differentiated the severity of injury better than MGAP (AUC = 0.801). CRAMS also discriminated well (AUC = 0.780), and T-TRS (AUC = 0.628) discriminated acceptably well. With a threshold value of 18, GAP had a sensitivity of 98.8% and a specificity of 42%. The MGAP trauma score, with a cut-off value of 22, also had a high sensitivity of 93.4% and had the best specificity at 62%. CRAMS, with a cut-off value of 8, had a sensitivity of 87.2% and a specificity of 40%. T-RTS had a similar sensitivity of 87.5% but had the lowest specificity.

**Figure 2:**
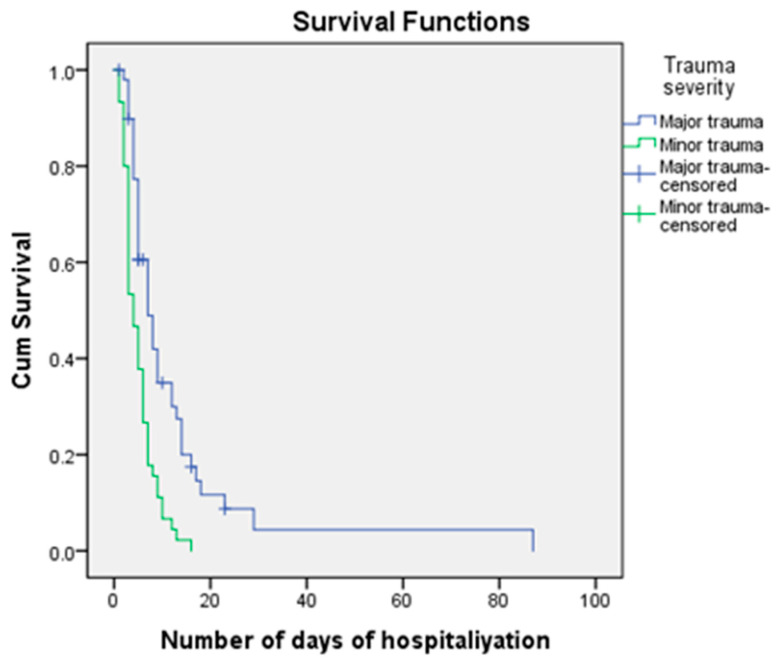
Kaplan–Meier curve according to severity of trauma.

Hospitalization of the patients with minor trauma (45/257; 17.5%) lasted from 1 to 16 days, and all patients survived. Patients with major trauma were hospitalized from 1 to 87 days, and no patients died after 23 days. Surviving patients with major trauma stayed in the hospital for significantly longer (*p* < 0.001). The median survival of patients with major trauma was 7 patient days (95% confidence interval = 4.674–9.326), while the median for patients with minor trauma was 4 patient days (95% confidence interval = 2.964–5.036) (Figure 2).

The calculated trauma scores were analyzed by using univariate regression. Taking minor trauma as a reference value, all trauma scoring systems were statistically significant in predicting the minor trauma (Table 4). We tested only two scores using multivariate regression analysis due to their strong correlation. Because there is a strong correlation between tRTS and CRAMS (r = 0.83), and MGAP and GAP (r = 0.95), we only analyzed CRAMS and MGAP using multivariate regression. Multivariate regression analysis showed that MGAP and GAP were independent predictors of trauma severity (OR 1.28; 95% Cl 1.05–1.49; *p* = 0.001). Neither the CRAMS scale nor tRTS were independent predictors of trauma severity (OR 0.55; 95% Cl 0.38–1.01; *p* = 0.054).

Taking the survival of hospitalized patients in the period up until discharge as a reference point in the univariate regression analysis, all four scoring systems were statistically significant (Table 5). However, it was shown that only MGAP (and GAP, with which MGAP is strongly correlated) was an independent predictor of survival of hospitalized patients (OR 2.23; 95% Cl 1.06–4.69; *p* = 0.035). With an increase in the MGAP score by 1, the probability of survival increased by 2.2 times.

## 4. Discussion

The aim of this study was to identify the trauma scoring system with the highest accuracy in evaluating trauma severity and predicting outcome. The older scoring systems were compared with newer ones in order to more precisely identify the 5% of critically injured and the additional 10–15% of patients who should be urgently treated in trauma centers [4,5]. The American College of Surgeons Committee on Trauma (ACS-COT) recommends that these groups of patients should be treated in primary and secondary trauma centers [12]. Based on the prehospital trauma scoring systems, it is possible to identify the severity of injuries and transport patients to the appropriate trauma centre. In addition to this, they also guide appropriate prehospital treatment, turning prehospital triage into a key step in the management of trauma patients [13,14]. Our study showed that almost half of major injuries were classified as minor ones by prehospital EMS doctors when asked to assess the trauma severity without using the scoring systems. After clinical diagnostics and the calculation of trauma scores, it was shown that these patients in fact had major injuries. It is necessary to use trauma scoring systems during the prehospital treatment of trauma patients in order to arrange transport to the appropriate trauma center level. If we identify a patient with minor trauma, they will be transported to a lower-level trauma centre. However, if a physician determines during the diagnostic test that a patient has major trauma, due to the lack of personnel and the appropriate equipment, the patient will be transported to a higher-level trauma centre. In that case, time from injury to definitive treatment of a trauma patient is prolonged, thereby reducing the chances of survival.

We tested four trauma scoring systems: the anatomical CRAMS scale and three physiological scoring systems. The CRAMS scale and RTS have been used for several decades, while MGAP and GAP were designed in the last fifteen years. The quality of each scoring system can be assessed not only by its sensitivity and specificity, but also by its predicative value. It is possible for a scoring system with low sensitivity to classify a major trauma as a minor one, thus leading to inadequate trauma management and transportation to an inappropriate trauma centre, ultimately resulting in higher mortality rates [13]. On the other hand, the low specificity of a scoring system can also result in minor traumas unnecessarily being treated as major ones, leading to a heavy workload at secondary trauma centers and resulting in delays in the management of major traumas [15]. In most trauma systems worldwide, a substantial number of severely injured patients are not transported to the appropriate level trauma centre [16].

The trauma score with the highest sensitivity and specificity in terms of patient treatment outcome is the ISS score. However, it is not suitable for use at the prehospital and initial hospital levels because it is an anatomical score that requires complex diagnostic tests [17]. At the prehospital and initial hospital levels, the most often used trauma score is RTS. Numerous studies have tested the sensitivity of the RTS score in assessing the severity of trauma, but mostly in hospital conditions. Its sensitivity value ranges between 59 and 83.5%, and its specificity from 82 to 94% [18,19]. However, although its sensitivity value when used at the prehospital level is similar to that at the hospital level (±70%), its measured specificity is significantly lower, reaching only 8.1% [20]. Given that high sensitivity and specificity are necessary in the assessment of the severity of trauma, new scores were tested and designed. An attempt was made to use anatomical trauma scores in the initial care of the injured, resulting in the CRAMS scale. When tested on data from 2019, it showed a sensitivity of 72% and specificity of 87%. However, when applied at the initial hospital level, the sensitivity and specificity of the CRAMS scale did not differ significantly from those of the RTS score [21]. Two newly developed physiological trauma scores, MGAP and GAP, have high sensitivity and specificity in identifying patients who will likely have an unfavorable outcome from treatment after trauma. According to some authors, the sensitivity of the MGAP score was measured at 93.7% and the specificity at 91.3%, while the sensitivity for predicting the unfavorable outcome of treatment using the GAP score ranged from 83.3 to 97.6%, and the specificity was 80 to 87% [22,23,24]. However, the use of these scores in identifying trauma severity is questionable. According to a 2018 study by Galvagno et al., the MGAP score has better sensitivity and specificity in terms of adverse treatment outcomes but is not superior in assessing the severity of trauma compared to the t-RTS score [25].

Our study tested the specificity and sensitivity of four trauma scoring systems in the prehospital setting. GAP had the highest sensitivity for the limit value (indicating major or critical trauma), but its specificity was lower compared to that of MGAP, although it was higher compared to tRTS and the CRAMS scale. MGAP had the best sensitivity/specificity ratio for the limit value. MGAP sensitivity was slightly above that of tRTS. However, its specificity was considerably higher than those of all other scoring systems, and so it was proven to be the best prehospital scoring system for assessing the severity of trauma. It is easy to apply and measure, and therefore it could find wide application by emergency medical teams at the prehospital level.

In addition to calculating the specificity and sensitivity of trauma scoring systems, we also used univariate regression analysis to determine the impact of scoring systems on the identification of trauma severity. All four prehospital scoring systems were statistically significant and were single predictors in assessing trauma severity. After the multivariate regression analysis, MGAP was singled out as an independent predictor of trauma severity, as was GAP, which strongly correlates with MGAP. The other two scoring systems were not statistically significant independent predictors for assessing trauma severity. 

All of the scoring systems were single predictors in the univariate regression analysis of treatment outcome. However, when the multivariate regression analysis was applied, MGAP was again identified as an independent predictor, together with the strongly correlating GAP. Similar results were obtained in recent studies. MGAP and GAP, tested in the initial period of the hospital setting, were found to be independent predictors, and no significant difference was found between the two [26]. In another study, RTS, ISS, TRISS (trauma and injury severity score), MGAP, and GAP were compared in their ability to predict mortality both within 24 h after admission and after four weeks. With the exception of RTS, all scoring systems could predict a fatal outcome and were statistically significant, but MGAP had the best predictive value [23]. In a study of 5484 trauma patients, MGAP was statistically significant in predicting the outcome of treatment [22]. A comparison of RTS, MGAP, and GAP in undeveloped countries with limited health resources showed that MGAP was superior in prehospital settings [27]. Compared with RTS, MGAP was superior in assessing trauma severity and predicting the outcome. MGAP performed worse than TRISS, although this scoring system can be applied only in a hospital setting after diagnosis is finished [28]. In a recent study, MGAP was found to be inferior to newly designed scoring systems, such as mREMS (modified rapid emergency medicine); however, this study also showed that MGAP was superior in comparison with RTS. MGAP and GAP were also shown to be better at predicting treatment outcome than NTS (new trauma score) [29,30]. A comparison between MGAP and GAP showed that there is no statistical significance between the two, meaning that both scoring systems can be used equally [31]. Furthermore, our study showed that these two scoring systems can be used both to assess the severity of the trauma and to predict a fatal outcome of the injured person’s treatment. With their use, patients would be transported to the correct trauma centers, where they would receive the best treatment in the shortest period of time. This would also avoid the overloading of trauma centers of the highest level with minor injury patients.

The limitation of this study was its small sample of patients. A large population of trauma patients is necessary to prove our conclusions. Our results refer to the prehospital level of trauma management, and not many trauma studies deal with the prehospital setting. Extensive research of trauma should be carried out at the prehospital level to prove our results. The organization of EMS, here, was important since each EMS team had a doctor present. It is unknown whether the results would be the same from teams where only paramedics work. It is also unknown whether the results would apply in rural areas, where hospitals are far away and transport time is significantly longer, or in conditions where a helicopter service is involved in the care of the traumatized and the transport time is significantly shorter. In addition, the data refer exclusively to the prehospital level. It is necessary to check the applicability of our results at the initial hospital level. In our study, we included adult men and women. It is necessary to check whether the same results would be obtained if men and women were observed separately, and especially whether they would also be applicable to children. We did not collect data on comorbidities or the use of drugs that could affect the value of SBP, HR, and RR, including oral anticoagulant drugs. Patients included in the study were not tested for alcohol or psychoactive substances, the use of which can significantly change GCS values.

## 5. Conclusions

MGAP and GAP scores have a higher sensitivity and specificity in the assessment of the severity of trauma, and both stand out as independent predictors in the assessment of trauma severity and the identification of injured persons who are at risk of an unfavorable treatment outcome after injury. They are easy to measure and do not require any additional diagnostics, providing reliable data on the patient’s condition. Their application would shorten the time of definitive treatment of the injured and help provide the most appropriate treatment, thus reducing the risk of death.

## Figures and Tables

**Table 1:** Average prehospital vital parameters in relation to trauma severity and outcome (mean ± SD).

	Age	RT	GCS	SaO_2_	HR
Minor trauma	52.03 ± 20.98	7.50 ± 4.26 ^‡,b^	14.80 ± 0.74 ^†,a^	97.33 ± 1.86 ^‡,a^	88.99 ± 15.74
Major trauma	49.86 ± 20.52	5.96 ± 2.92	12.00 ± 3.59	91.26 ± 11.04	90.02 ± 17.44
Survived	51.41 ± 20.87	7.27 ± 4.14	14.55 ± 1.33 ^‡,a^	96.92 ± 2.93 ^‡,a^	89.19 ± 15.58
Fatal outcome	61.38 ± 20.26	6.38 ± 2.67	6.63 ± 3.42	75.00 ± 20.32	88.00 ± 29.26
Total	51.67 ± 20.88	7.25 ± 4.11	14.35 ± 1.90	96.35 ± 5.24	89.16 ± 16.00

SD—standard deviation; ^†^—*t*-test; ^‡^—Mann–Whitney U test; ^a^—*p* < 0.001; ^b^—*p* < 0.01; GCS—Glasgow Coma Scale; SaO_2_—blood oxygen saturation; HR—heart rate; RT—EMS response time.

**Table 2:** Average trauma scores, according to the categories (mean ± SD).

	CRAMS	T-RTS	MGAP	GAP
Minor trauma	9.55 ± 0.86 ^a^	11.81 ± 0.58 ^a^	26.21 ± 2.68 ^a^	22.17 ± 1.80 ^a^
Major trauma	8.12 ± 1.82	11.12 ± 1.52	21.30 ± 5.09	18.00 ± 4.05
Survived	9.41 ± 1.03 ^a^	17.70 ± 0.43 ^a^	25.75 ± 3.04 ^a^	21.75 ± 2.24 ^a^
Fatal outcome	5.88 ± 1.88	5.39 ± 1.70	13.00 ± 3.74	11.75 ± 3.66
Total	9.32 ± 1.20	11.70 ± 0.84	25.41 ± 3.66	21.48 ± 2.78

SD—standard deviation; mean; ^a^—*p* < 0.001; CRAMS scale—circulation, respiration, abdomen, motor, and speech; T-RTS score—revised trauma score for triage; MGAP—mechanism, Glasgow Coma Scale, age, systolic blood pressure; GAP—Glasgow Coma Scale, age, systolic blood pressure.

**Table 3:** ROC analysis of T-RTS, CRAMS, MGAP, and GAP trauma scores for injury severity.

	T-RTS	CRAMS	MGAP	GAP
AUC	0.628	0.780	0.801	0.842
*p*	0.004	0.000	0.000	0.000
95% CI	0.534–0.722	0.707–0.853	0.725–0.878	0.771–0.910
Cut-off	11	8	22	18
Se(%)	87.5	87.2	93.4	98.8
Sp(%)	36	40	62	42
PPV	0.727	0.714	0.857	0.875
NPV	0.858	0.878	0.888	0.898
LR^+^	1.289	1.453	2.458	1.703
LR^−^	0.347	0.320	1.064	0.029

ROC—receiver operating characteristic; AUC—area under the curve; *p*—statistical significance; 95% CI—95% confidence interval; Se—sensitivity; Sp—specificity; PPV—positive predictive value; NPV—negative predictive value; LR^+^—positive likelihood ratio; LR^−^—negative likelihood ratio; CRAMS scale—circulation, respiration, abdomen, motor, and speech; T-RTS score—revised trauma score for triage; MGAP—mechanism, Glasgow Coma Scale, age, systolic blood pressure; GAP—Glasgow Coma Scale, age, systolic blood pressure.

**Table 4:** Univariate and multivariate regression analysis of prehospital trauma scoring systems in relation to trauma severity.

	Univariate Analysis			Multivariate Analysis		
	*p*	OR	95% CI	*p*	OR	95% CI
T-RTS	0.000	2.14	1.52–3.01			
CRAMS	0.000	2.30	1.75–3.02	0.054	0.53	0.38–1.02
MGAP	0.000	1.51	1.33–1.72	0.001	1.28	1.05–1.49
GAP	0.000	1.89	1.56–2.3			

Reference range—minor trauma; *p*—statistical significance; OR—likelihood ratio; 95% CI—95% confidence interval; CRAMS scale—circulation, respiration, abdomen, motor, and speech; T-RTS score—revised trauma score for triage; MGAP—mechanism, Glasgow Coma Scale, age, systolic blood pressure; GAP—Glasgow Coma Scale, age, systolic blood pressure; bold values are statistically significant.

**Table 5:** Univariate and multivariate regression analysis of prehospital trauma scoring systems in relation to outcome.

	Univariate Analysis			Multivariate Analysis		
	*p*	OR	95% CI	*p*	OR	95% CI
RTS	0.002	4.85	1.81–13.01			
CRAMS	0.003	2.19	1.31–3.65			
MGAP	0.001	1.28	1.05–1.49	0.035	2.23	1.06–4.69
GAP	0.002	1.88	1.27–2.80			

Reference range—survived; *p*—statistical significance; OR—likelihood ratio; 95% CI—95% confidence interval; CRAMS scale—circulation, respiration, abdomen, motor, and speech; T-RTS score—revised trauma score for triage; MGAP—mechanism, Glasgow Coma Scale, age, systolic blood pressure; GAP—Glasgow Coma Scale, age, systolic blood pressure; bold values are statistically significant.

## Data Availability

Not applicable.
